# Distribution of macrophages and plasma cells in apical periodontitis and their relationship with clinical and image data

**DOI:** 10.4317/jced.53758

**Published:** 2017-09-01

**Authors:** Stéphane V. Azeredo, Sabrina C. Brasil, Henrique Antunes, Fábio V. Marques, Fábio R. Pires, Luciana Armada

**Affiliations:** 1 DDS, MSc, Department of Endodontics, Faculty of Dentistry, Estácio de Sá University; 2 DDS, MSc, PhD, Department of Endodontics, Faculty of Dentistry, Estácio de Sá University

## Abstract

**Background:**

Macrophages and plasma cells play a key role in the regulation of innate and adaptive immunity. The aim of this study was to assess the presence of these cells in apical periodontitis and their distribution comparing with clinical and image data.

**Material and Methods:**

Thirty-three lesions were selected and divided in two groups (17 periapical cysts and 16 periapical granulomas). Immunoreactions using anti-CD68 and anti-CD138 antibodies were carried out; image analysis was performed with an optical microscope and 5 high-power fields from each slide were evaluated leading to an average score of immunoexpression. This mean score was compared between the two groups and correlated with the clinical and image data.

**Results:**

There was no statistically significant difference (*p* >0.05) for the mean average score of CD68+ macrophages and CD138+ plasma cells when comparing the two groups (cysts x granulomas) and the specimens included in each specific group. No statistically significant differences (*p* >0.05) were also observed when comparing the average scores with clinical and image data.

**Conclusions:**

The presence of CD68+ macrophages and CD138+ plasma cells was similar in periapical cysts and granulomas and the presence of these cells did not correlate with clinical and image data from both groups.

** Key words:**Macrophages, plasma cells, apical periodontitis, periapical granuloma, periapical cyst.

## Introduction

Apical periodontitis (AP) is the main response of periradicular tissues to a variety of stimuli that cause pulpal and periradicular damage (1). In periapical granulomas and cysts, tissue destruction by host defense mechanisms seems to be more significant than the direct effects of microbial products being discharged from the infected root canal, even if the microorganisms have triggered the whole periradicular inflammation process (2,3).

Macrophages are frequently found in AP (4-10) and because of the wide range of pro-inflammatory and anti-inflammatory mediators, as well as substances that enhance tissue repair, produced by these cells, they have a fundamental role in the formation, perpetuation and involution of AP. Plasma cells are also present in AP (6-10) and their main products of secretion - immunoglobulins - seem to be directly involved in the immune response against intraradicular microbial attack and in the stabilization of these lesions.

Although the presence of macrophages and plasma cells in AP is well-established, their possible association with clinical and imaging features is not clear. Therefore, the aim of this study was to assess the presence of macrophages and plasma cells in periapical granulomas and cysts, comparing the results with clinical and image data.

## Material and Methods

-Sample selection

The files of the Oral Pathology laboratory, Estácio de Sá University, were reviewed and paraffin blocks presenting sufficient material for histological and immunohistochemical analysis and containing clinical and radiological information were retrieved. All specimens were obtained through periradicular surgery (persistent or secondary intraradicular infections), and had been performed under local anesthesia and following the same protocol of Bracks (10). The curetted lesions were fixed in 10% buffered formalin for 48 hours, processed for routine histological analysis and stained with hematoxylin and eosin. Histological diagnoses were carried out by two calibrated examiners. All cases presented complete clinical (age, gender, location of the lesion, presence of symptoms and presence of sinus tract) and tomographic (size of the lesion, measured by its largest diameter and classified as small when < 5 mm or large when ≥ 5 mm) data information.

Patients with metabolic and immunosuppressive diseases such as diabetes and infection with the human immunodeficiency virus, as well as autoimmune diseases, were excluded from the work. Patients with insufficient imaging and clinical records, and biopsy specimens too small for histological analysis were also excluded. Participants were informed about the nature of the study and signed an informed consent form. The research project was approved by the Ethics Committee of the University Estacio de Sá (CAAE: 47669715200005284).

-Immunohistochemistry

For the immunohistochemical analysis, 3-µm tissue sections from the paraffin-embedded blocks were mounted on 3-aminopropyltriethoxy-silane coated glass slides (Sigma Chemical Co., St Louis, MO), deparaffinize with xylene and rehydrated in graded alcohol. The slides were then immersed in citrate buffer, pH 6.0, for 1 cycle of 12 minutes in a microwave oven for antigen retrieval. After this step, the slides were submitted to 5 consecutive 6% hydrogen peroxide 5-minute baths, and left in phosphate-buffered saline (PBS) 1×, pH 7.4 (Laborclin, Pinhais, PR, Brazil). The slides were incubated with primary mouse monoclonal antibodies anti-CD68 (1:500, M0876, DAKO North America, Carpinteria, CA, USA) and anti-CD138 (1:100, M7228, DAKO North America, Carpinteria, CA, USA) at 4oC overnight in a humidified chamber. After this period, the slides were washed in PBS and the sections were treated with a labeled streptavidin-biotin kit (LSAB, DAKO North America, Carpinteria, CA, USA). Peroxidase activity was visualized by immersing tissue sections in 3,3’-diaminobenzidine (DAB, DAKO North America, Carpinteria, CA, USA), counterstained with Mayer’s hematoxylin. Positive controls for CD68 (Mucocele) and CD138 (Inflammatory gingival hyperplasia) and negative controls (omission of primary antibodies) were used for all reactions.

-Image Analysis

The presence of CD68+ macrophages and CD138+ plasma cells were independently analyzed by two experienced observers using light microscopy. After analyzing the whole specimen, five high power fields containing connective tissue and/or epithelium, from each sample were evaluated and the expression of each marker was classified according to the following scores: 0 (negative/focal), if there were no positive cells or less than 5% of the cells were positively stained; 1 (weak to moderate) if between 5% and 50% of the cells were positively stained; and 2 (strong), if more than 50% of the cells were positive. The average score for the entire sample was calculated, and the expression of CD68+ macrophages and CD138+ plasma cells were classified as negative/focal (final average from 0 to 0.5), weak to moderate (between 0.6 and 1.2) and strong (ranging from 1.3 to 2.0).

-Statistical analysis 

Data analysis was performed using the Mann-Whitney test when comparing cysts and granulomas; Fisher’s exact test was used to evaluate the presence of symptoms and sinus tract and demographic parameters (except age); Mann-Whitney test was used to compare mean age and lesion size. Statistical significance was considered when *p*<0.05.

## Results

A total of 33 lesions (17 periapical cysts and 16 periapical granulomas) were selected. Age of the patients ranged from 15 to 78 years old and the mean ages of the patients affected by cysts (44.5 ± 15.7 years old) and granulomas (47.3 ± 14.0 years old) were similar (*p*>0.05). Females represented 58.8% and 50% of the patients affected by cysts and granulomas, respectively (*p*>0.05); Cysts were mostly located in the maxilla (14 cases, 82.4%) and in the anterior region (13 cases, 76.5%), similarly to granulomas (13 cases, 81.3% in the maxilla; 11 cases, 68.8% in the anterior region) (*p*>0.05, both). None of the cysts was symptomatic, while 6 (38%) of the granulomas presented with symptoms (*p*=0.007). Sinus tract was present in 11 cysts (65%) and in 5 granulomas (32%) (*p*=0.08). The mean greatest tomographic diameter of the cysts (11.9 ± 5.2 mm) was greater than the mean greatest tomographic diameter of the granulomas (7.9 ± 4.6 mm) (*p*=0.009).

**Figure 1:**
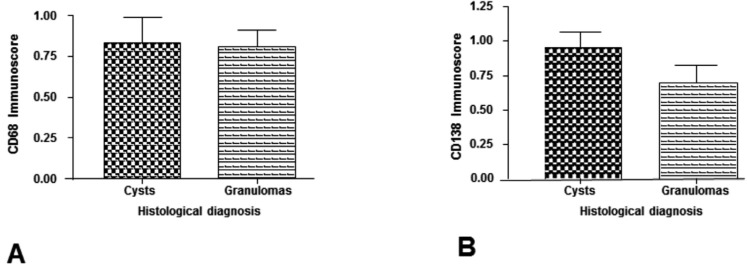
Comparison between periapical cysts and granulomas. A: CD68. B: CD138. (Mann-Whitney test) (*p*<0.05).

**Figure 2:**
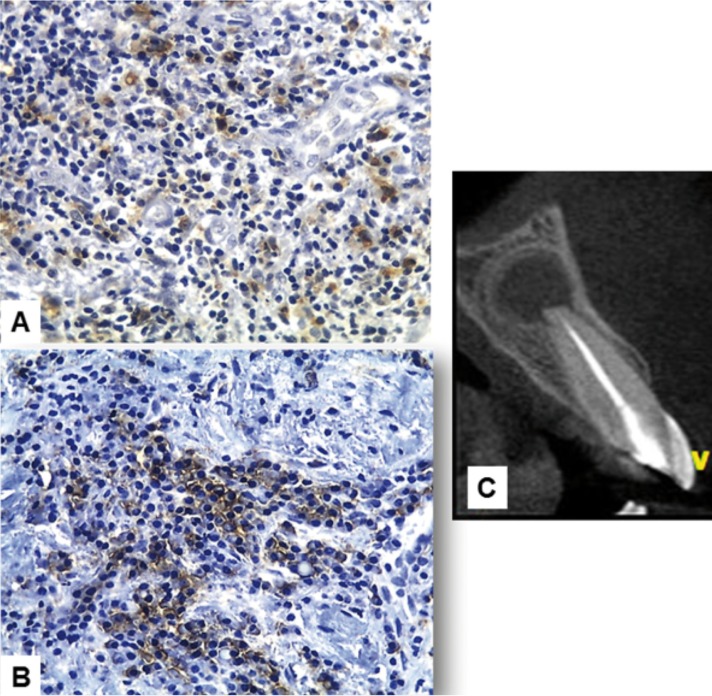
Periapical cyst. A: CD68+ macrophages. B: CD138+ plasma cells (immunoperoxidase, original magnification, x400). C: tomographic image.

**Figure 3:**
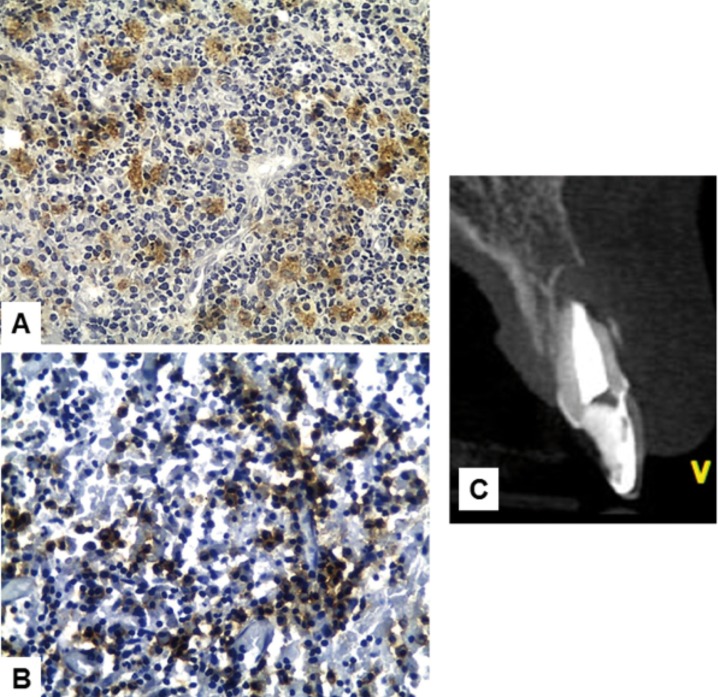
Periapical granuloma. A: CD68+ macrophages. B: CD138+ plasma cells (immunoperoxidase, original magnification, x400). C: tomographic image.

There was no statistically significant difference when comparing the mean score of CD68+ macrophages in cysts (0.83 ± 0.63) and granulomas (0.81 ± 0.39) (*p*>0.05) (Fig. 1A). There was also no statistically significant difference when comparing the mean average score of CD138+ plasma cells in cysts (0.95 ± 0.46) and granulomas (0.70 ± 0.49) (*p*>0.05) (Fig. 1B).  Table 1 shows the distribution of the immunoscore of CD68+ macrophages and CD138+ plasma cells in periapical cysts and granulomas. There was no statistical significant differences whe comparing clinical (age, gender, and presence of symptoms and sinus tract) and image (CBCT mean greater diameter) data with both CD68 and CD138 immunoscores (*p*>0.05) ( Table 2). Figures 2 and 3 show the distribution of CD68 + macrophages and CD138+ plasma cells and CBCT scans of a periapical cyst and a periapical granuloma.

**Table 1:**
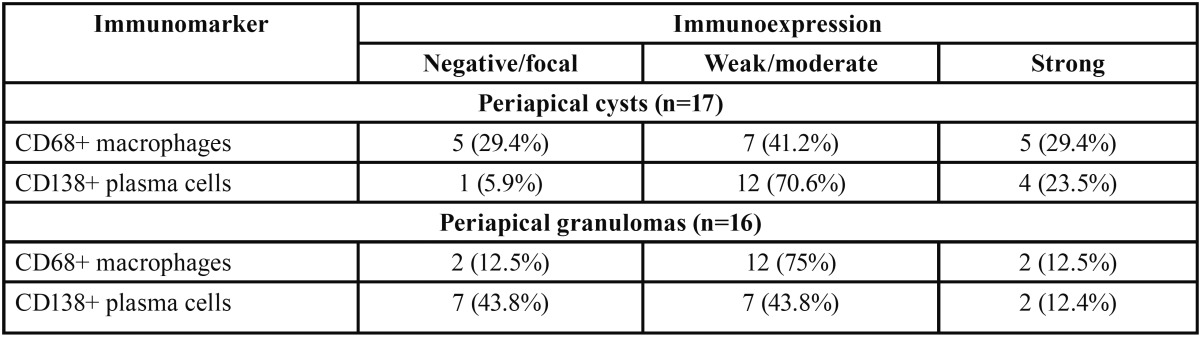
Distribution of the immunoscore of CD68+ macrophages and CD138+ plasma cells in periapical cysts and granulomas.

**Table 2:**
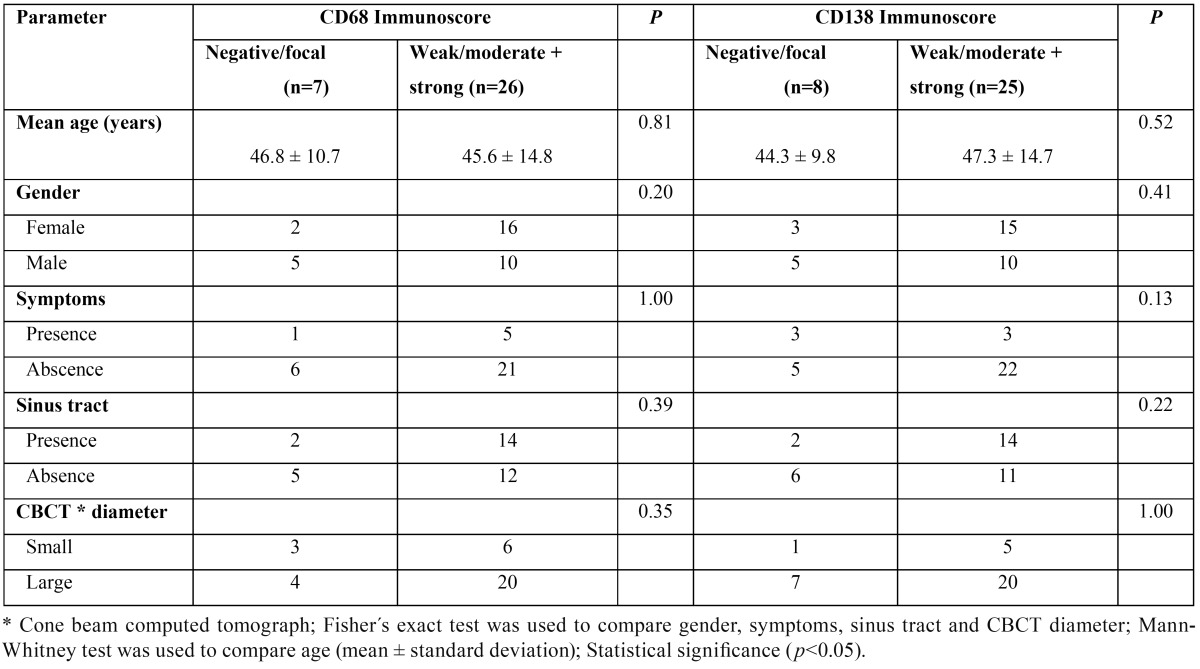
Distribution of clinical and image parameters according with CD68 and CD138 immunoscores.

## Discussion

In the periradicular region, the interaction between inflammatory cells and microorganisms and their byproducts result in specific and non-specific immune responses. Various studies have carried out investigations into the quantitative analysis of immunocompetent cells involved in AP (7-13). The present results support that macrophages and plasma cells are common components of the inflammatory infiltrate in both periapical cysts and granulomas.

There was no significant difference in the mean immunoscore of CD68+ macrophages in cysts and granulomas, which is in accordance with the results of Rodini and Lara (8). There was also no significant difference between the proportion of macrophages and plasma cells in these lesions. These findings differ from the results obtained by Stern et al. (14) who found that macrophages are the predominant inflammatory cells, followed by lymphocytes, plasma cells and neutrophils and Lukic et al. (11) who found that most inflammatory cells in periapical granulomas and cysts were lymphocytes and plasma cells, followed by macrophages. According to Metzger (15), the presence of macrophages in AP is well understood, and these cell types can be considered the main constituents of granulomas.

According to Akamine *et al.* (6), macrophages had a close relationship with bone destruction and plasma cells could participate in tissue repair rather than lesion development. Based on these findings, we suggest that this similarity of cells counts between periapical granulomas and cysts is related with controls the extent and outcome of the host response to antigen stimulation during chronic inflammatory processes.

Our results demonstrated the intense staining of the CD138+ plasma cells in cysts and granulomas, corroborating previous studies (16,17). However, there was no statistically significant difference when comparing the mean score of CD138+ plasma cells in cysts and granulomas.These findings are in agreement with the data obtained by Liapatas *et al.* (9).

Our results did not show any statistically significant difference between the symptomatic and asymptomatic lesions with respect to the proportion of macrophages and plasma cells, in accordance with previous studies (11). No statistically significant differences were also observed when analyzing the presence of sinus tract.

The mean greatest diameter of periapical cysts was statistically significantly greater than that of the periapical granulomas in the present work. These findings reinforce the most accepted concept for the pathogenesis of periapical cysts, which are lesions originated from granulomas, that supposedly have a longer evolution time (18,19). Our results did not demonstrated any significant differences between on CD68+ macrophages and CD138+ plasma cells immunoscores when comparing large and small lesions, diverging from the results of Gazivoda *et al.* (20) who found that larger lesions contained a lower percentage of mononuclear phagocytes compared to smaller lesions. The results from this study showing no differences may be due to the low number of samples used, differences of image analysis or time of lesion development.

In conclusion, the present study confirms the involvement of macrophages and plasma cells in the inflammatory process of AP and suggests that this similarity of cells counts between periapical granulomas and cysts is related with controls the extent and outcome of the host response to antigen stimulation during chronic inflammatory processes. There were no differences on the presence of these cells according with clinical and image parameters.
